# Synthesis and characterization of supported stabilized palladium nanoparticles for selective hydrogenation in water at low temperature†

**DOI:** 10.1039/d1ra00239b

**Published:** 2021-02-19

**Authors:** Anish Patel, Anjali Patel

**Affiliations:** Polyoxometalates and Catalysis Laboratory, Department of Chemistry, Faculty of Science, The Maharaja Sayajirao University of Baroda Vadodara-390002 Gujarat India anjali.patel-chem@msubaroda.ac.in

## Abstract

Zirconia supported vacant phosphotungstate stabilized Pd nanoparticles (Pd–PW_11_/ZrO_2_) were prepared using a simple impregnation and post reduction method, characterized and their efficiency for selective C=C hydrogenation of unsaturated compounds explored. The establishment of a hydrogenation strategy at low temperature using water as solvent under mild conditions makes the present system environmentally benign and green. The catalyst shows outstanding activity (96% conversion) with just a small amount of Pd(0) (0.0034 mol%) with high substrate/catalyst ratio (29 177/1), TON (28 010) and TOF (14 005 h^−1^) for cyclohexene (as a model substrate) hydrogenation. The catalyst was recovered by simple centrifugation and reused for up to five catalytic cycles without alteration in its activity. The present catalyst was found to be viable towards different substrates with excellent activity and TON (18 000 to 28 800). A study on the effect of addenda atom shows that the efficiency of the catalyst can be enhanced greatly by increasing the number of counter protons. This challenging strategy would greatly benefit sustainable development in chemistry as it diminishes the use of organic solvents and offers economic and environmental benefits as water is cheap and non-toxic.

## Abstract 2

Zirconia supported vacant phosphotungstate stabilized Pd nanoparticles (Pd–PW_11_/ZrO_2_) are proved to be sustainable and excellent for the water mediated hydrogenation reaction with a very high substrate to catalyst ratio, TON and TOF.

## Graphical abstract

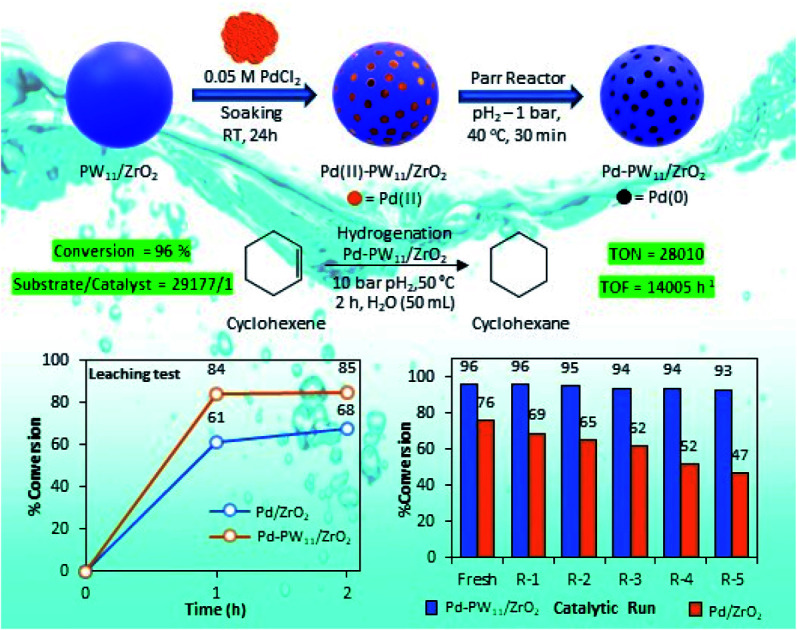

## Introduction

Palladium nanoparticles (PdNPs) are one of the most studied catalysts for organic transformations and historically dominant over other metals, and they still serve as a central tool for innumerable important organic transformations and total synthesis.^1^ Nanosizing of Pd based bulk catalysts is an emerging and promising field owing to the allowance of Pd's maximum utilization, and enhanced efficiency, activity and selectivity.^2^ Particle size is a key factor of catalytic performance: by decreasing the metal size, the specific activity can be increased greatly^3^ (Fig. S1†). However, due to high surface energy, PdNPs are very much prone to form aggregates during either their synthesis or use in catalytic reactions, and hence, stabilization of PdNPs is necessary.^4^

Number of efforts have been made for the same using different stabilizing agents such as dendrimers,^5^ phosphine base ligands,^6^ surfactants,^7^ ionic liquids,^8^*etc.* As the reported stabilizing scaffolds are mostly organic, toxic and expensive, it is important to replace the same by some alternatives. Heteropoly acids (HPAs) are the excellent candidate for the same. HPAs are discrete early transition metal–oxide cluster anions and comprise a class of inorganic complexes^9^ having unrivalled versatility and structural variation in both symmetry and size.^10^ In addition, they have following advantages: (i) Robust oxoanionic nature which greatly enhance its stability power, (ii) reducing capacity, favours the stability of Pd in to its most stable oxidation state zero,^11^ (iii) large relative sizes which sterically hindered PdNPs and prevent it from agglomeration during the synthesis as well as its catalytic reaction and (iv) it avoids the use of external ligands for stabilization of PdNPs, which are mostly organic-toxic materials.

Liquid-phase selective hydrogenation is a diverse and versatile acceptable route to synthesize precursors for various intermediates and still it is fascinating and challenging field.^12^ In this regards, triethanolamine,^13^ isopropanol^14^ and oxalic acid^15^*etc.*, are generally used as electron and proton donors for hydrogenation, which makes the process less sustainable. Moreover, the processes which involves the use of molecular hydrogen are usually suffer from harsh reaction conditions, lower selectivity^16^ and use of high active amount of the catalyst.^12*d*^ In advancement, it is highly attractive to design a green strategy for direct utilization of the molecular hydrogen using water as solvent under mild reaction conditions.

In this context, our previous report^12*d*^ demonstrates the use of zirconia supported 12-tungstophosphoric acid (Pd–TPA/ZrO_2_) as stabilizing scaffold and its outstanding activity towards hydrogenation of different aromatic and aliphatic compounds inspired us to design another catalyst. Our main objective is to develop the catalyst having upgraded activity for water mediated hydrogenation with high substrate/catalyst ratio and low active amount of Pd under mild reaction conditions. Hence, in present case we have selected mono lacunary tungstophosphoric acid (PW_11_) supported on zirconia (ZrO_2_) as a stabilizing agent. The selection of PW_11_ was the eye-catchy step as it has seven counter protons (higher than the parent one, TPA = PW_12_) which can accelerate the formation of Pd–H to enhance the hydrogenation rate.

Herein, first time we present a simple engineering method for PdNPs, based on supported mono lacunary tungstophosphoric acid (PW_11_/ZrO_2_) as effective stabilizing agent. Synthesized catalyst (Pd–PW_11_/ZrO_2_) was characterized by different spectroscopy techniques and sole presence of PdNPs was exposed by HRTEM and dark/bright field STEM. The efficiency of the catalyst was evaluated for the hydrogenation of cyclohexene as a model substrate using molecular hydrogen and water as solvent. The influence of various reaction parameters like catalyst amount, reaction time, temperature, pressure and solvent were studied deeply. Scope and limitations of the catalyst was also evaluated for various aromatic and aliphatic. The stability of the catalyst was confirmed by characterization of regenerated catalyst by EDX, FT-IR, XRD, XPS and TEM. The competence of the catalyst was compared with reported catalytic systems. Further, in order to understand the role of addenda atom, the catalytic activity was compared with Pd–PW_12_/ZrO_2_ (Pd–TPA/ZrO_2_) and explained on the bases of available protons. Mechanistic investigation was also studied by using D_2_O as solvent.

## Experimental

### Materials

Anhydrous disodium hydrogen phosphate, sodium tungstate dihydrate, acetone, nitric acid, hydrochloric acid, zirconium oxychloride, 25% (v/v) ammonia, palladium chloride, cyclohexene and dichloromethane were used as received from Merck without further purification. All chemicals used were of analytical reagent grade.

### Catalyst synthesis

Stabilized PdNPs *via* supported mono lacunary tungstophosphoric acid (Pd–PW_11_/ZrO_2_) was synthesized by wet chemistry method in two steps.

#### Step-1: Synthesis of supported mono lacunary tungstophosphoric acid (PW_11_/ZrO_2_) by incipient wet impregnation method

**Fig. 1:**
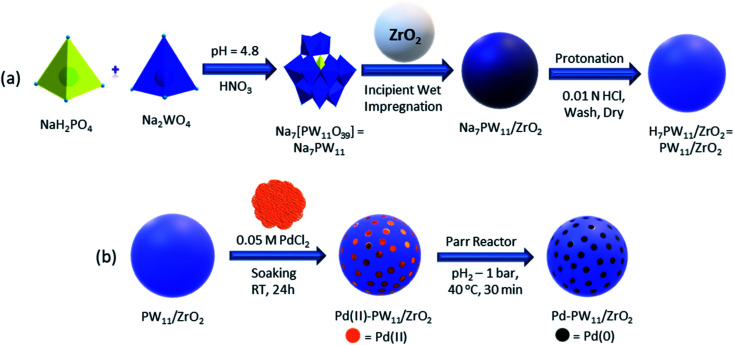
Synthesis of (a) PW_11_/ZrO_2_ and (b) Pd–PW_11_/ZrO_2_.

Sodium salt of mono lacunary tungstophosphoric acid^17^ (Na_7_PW_11_O_39_, later Na_7_PW_11_), zirconia^18^ (ZrO_2_) and PW_11_/ZrO_2_ (ref. 19) were synthesized following the methods reported by Brevard *et al.* and our group, respectively (for detail, see ESI†) as shown in Fig. 1a.

#### Step-2: Synthesis of Pd–PW_11_/ZrO_2_ by soaking and post reduction method

Palladium was deposited on PW_11_/ZrO_2_*via* exchanging the available protons of PW_11_ following the same method as reported earlier by our group for Pd–PW_12_/ZrO_2_ synthesis,^11^ which can be used for any polyoxometalate based material. In synthesis, 1 g of PW_11_/ZrO_2_ was soaked with 25 mL of 0.05 M aqueous solution of PdCl_2_ for 24 h with stirring. The solution was filtered, washed with distilled water in order to remove the excess of PdCl_2_ and dried in air at room temperature. The resulting (brown colored) material was designated as Pd(ii)–PW_11_/ZrO_2_. Finally, the synthesized material was charged into the Parr reactor under 1 bar H_2_ pressure, at 40 °C for 30 min to reduce Pd(ii) to Pd(0). The obtained (black colored) material was designated as Pd–PW_11_/ZrO_2_. The synthetic scheme is presented in Fig. 1b. The same procedure was followed for the synthesis of Pd/ZrO_2_ (for detail, see ESI†).

### Characterization

The synthesized materials were characterized by different physico-chemical techniques which are generally used for nanomaterials such as EDX, TGA, FT-IR, BET, XRD, XPS, TEM, HRTEM and BF/DF-STEM. The instrumental details are included in ESI.†

### Hydrogenation

The catalytic hydrogenation was carried out using Parr reactor as described in our recent article.^20^ In typical experiment, cyclohexene with water as solvent and catalyst were charged into the reactor vessel. The reactor was flushed thrice with H_2_ gas to remove the air present in the empty part of the vessel. Finally, H_2_ pressure was applied for the reaction. The reaction was set at desired temperature with the stirring rate of 1700 rpm for 2 h. The continuous decrease in pressure inside the vessel was utilized for determination of the reaction progress. After reaction completion, the reaction mixture was cooled at room temperature and H_2_ pressure was released from the vent valve. The organic layer was extracted by dichloromethane, whereas catalyst was collected from the junction of the liquid phases and finally recovered by centrifugation. The organic phases were dried with anhydrous magnesium sulfate and analyzed by a gas chromatograph (Shimadzu-2014) using a capillary column (RTX-5). The products were recognized by comparison with the standard samples.

## Results and discussion

### Characterization

**Fig. 2:**
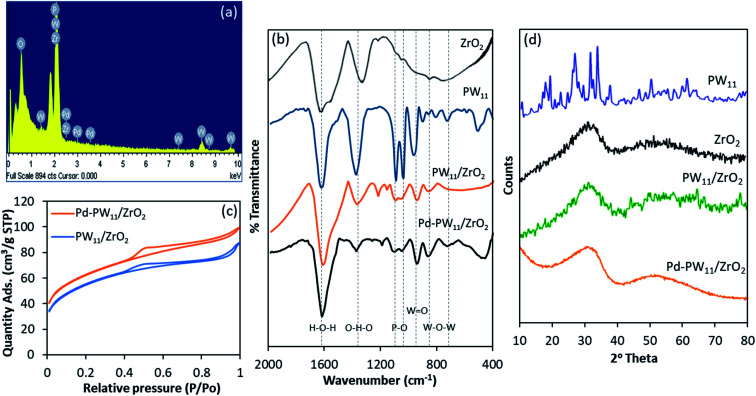
(a) EDX elemental mapping of Pd–PW_11_/ZrO_2_; (b) FT-IR spectra; (c) N_2_ sorption isotherms and (d) XRD spectra.

The gravimetric analysis of standard solution and filtrate showed 6.05 wt% and 0.72 wt% of Pd in Pd/ZrO_2_ and Pd(ii)–PW_11_/ZrO_2_, respectively.^21^ For Pd–PW_11_/ZrO_2_, EDX values of W (15.57 wt%) and Pd (0.76 wt%) are in good agreement with calculated one (15.19 wt% of W, 0.72 wt% of Pd). EDX elemental mapping of Pd–PW_11_/ZrO_2_ is shown in Fig. 2a.

Thermal stability of Pd–PW_11_/ZrO_2_ was evaluated by TGA and the obtained curve is plotted in Fig. S2.† Curve indicates 8.13% weight loss in the temperature range of 50 to 110 °C due adsorbed water molecule. Alongside, there was no significant weight loss observed up to 500 °C indicating the high thermal stability of the material.

The FT-IR spectra of ZrO_2_, PW_11_, PW_11_/ZrO_2_, Pd–PW_11_/ZrO_2_ are shown in Fig. 2b. ZrO_2_ shows broad bands in the region of 1600, 1370, and 600 cm^−1^ attributed to H–O–H and O–H–O bending and Zr–OH bending, respectively. FT-IR spectrum of PW_11_ exhibits bands at 1088, 1042, 964, 903 and 810 cm^−1^ corresponding to P–O, W=O and W–O–W stretching, respectively. Here, the splitting of P–O bond is due to the lowering of symmetry around central hetero atom phosphorus, indicates the formation of lacunary species.

Similarly, PW_11_/ZrO_2_ exhibits bands at 1090, 1047, 964 and 812 cm^−1^ corresponding to P–O, W=O, and W–O–W stretching vibration frequencies, respectively. No significant change in the bands indicate the retention of the Keggin unit in the synthesized material. The spectrum of Pd–PW_11_/ZrO_2_ shows bands at 1092, 1049, 960 and 806 cm^−1^ corresponding to P–O, W=O and W–O–W, respectively. The slight shift in the bands may be due to the change in environment by Pd.

The BET surface area is the useful tool to probe the quality and character of solid phase materials. Specific surface area of ZrO_2_, PW_11_/ZrO_2_, Pd(ii)–PW_11_/ZrO_2_ and Pd–PW_11_/ZrO_2_ were measured to investigate the chemical interaction between support and active species. The surface area of PW_11_/ZrO_2_ (224 m^2^ g^−1^) is found to be higher than support ZrO_2_ (170 m^2^ g^−1^). This is because of bulky anionic nature of PW_11_.^19^ The decrease in surface area of Pd(ii)–PW_11_/ZrO_2_ (199 m^2^ g^−1^) compared to PW_11_/ZrO_2_ indicates the strong interaction of Pd with the loaded material. The drastic rise in surface area of Pd–PW_11_/ZrO_2_ (213 m^2^ g^−1^) compared to Pd(ii)–PW_11_/ZrO_2_ is the first evidence for the presence of Pd(0) nanoparticles (PdNPs), due to the downsizing of Pd during the reduction of Pd(ii) to Pd(0). In spite of having different surface area, the unaltered nature of the N_2_ sorption isotherms of PW_11_/ZrO_2_ and Pd–PW_11_/ZrO_2_ (Fig. 2c) indicate the identical basic structure, the same is also reflected by FT-IR analysis.

To study the surface morphology and rate of dispersion in synthesized materials, the XRD patterns of PW_11_, ZrO_2_, PW_11_/ZrO_2_ and Pd–PW_11_/ZrO_2_ were recorded (Fig. 2d). XRD patterns of PW_11_ shows the characteristic peaks between 2*θ* range of 20° to 35°. Whereas, the absence of all patterns corresponds to PW_11_ in PW_11_/ZrO_2_ indicates the uniform dispersion over the surface of the support. XRD patterns of Pd–PW_11_/ZrO_2_ did not reflected any diffraction corresponds to PW_11_ as well as Pd, indicates the high degree of dispersion over the surface as well as no sintering of Pd was form during the synthesis.

**Fig. 3:**
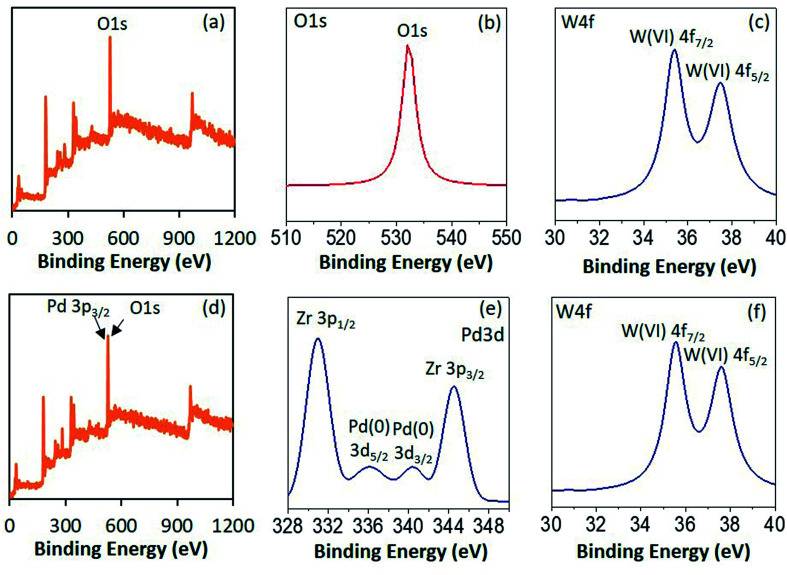
XPS spectra of PW_11_/ZrO_2_ (a–c) and Pd–PW_11_/ZrO_2_ (d–f).

To confirm the electronic state of the Pd, W and O high resolution XPS of PW_11_/ZrO_2_ and Pd–PW_11_/ZrO_2_ were recorded (Fig. 3). PW_11_/ZrO_2_ shows (Fig. 3 and b) a very intense peak at binding energy 532 eV corresponds to O 1s as it contains number of O atoms of PW_11_ and ZrO_2_ support, whereas Pd–PW_11_/ZrO_2_ shows (Fig. 3d) the direct overlap peak between Pd 3p_3/2_ and O 1s peaks at binding energy 532 eV, which is in good agreement with the reported one,^22^ and cannot be assigned to confirm the presence of Pd(0). Hence, we have presented instrument generated full spectra (Fig. 3a and d) images, supporting the presence of Pd(0). This is further confirmed by recording the high resolution Pd 3d spectrum which (Fig. 3e) shows a low intense spin orbit doublet peak at binding energy 335.9 eV and 340.5 eV correspond to Pd 3d_5/2_ and Pd 3d_3/2_, confirming the presence of Pd(0).^23^ Two additional high intense peaks at binding energy 331 eV and 345 eV attributed to Zr 3p_3/2_ and Zr 3p_1/2_, respectively^24^

PW_11_/ZrO_2_ shows (Fig. 3c) a well resolved spin–orbit doublet of W 4f_7/2_ and W 4f_5/2_ at binding energy 35.6 and 37.6 eV (spin–orbit splitting, 2.0 eV), characteristic of W(vi), confirming the presence of W(vi). Pd–PW_11_/ZrO_2_ also shows (Fig. 3f) a single spin–orbit pair at binding energy 35.6 and 37.5 eV (spin–orbit splitting, 1.9 eV) confirming no reduction of W(vi) during the synthesis.^23*b*,25^

**Fig. 4:**
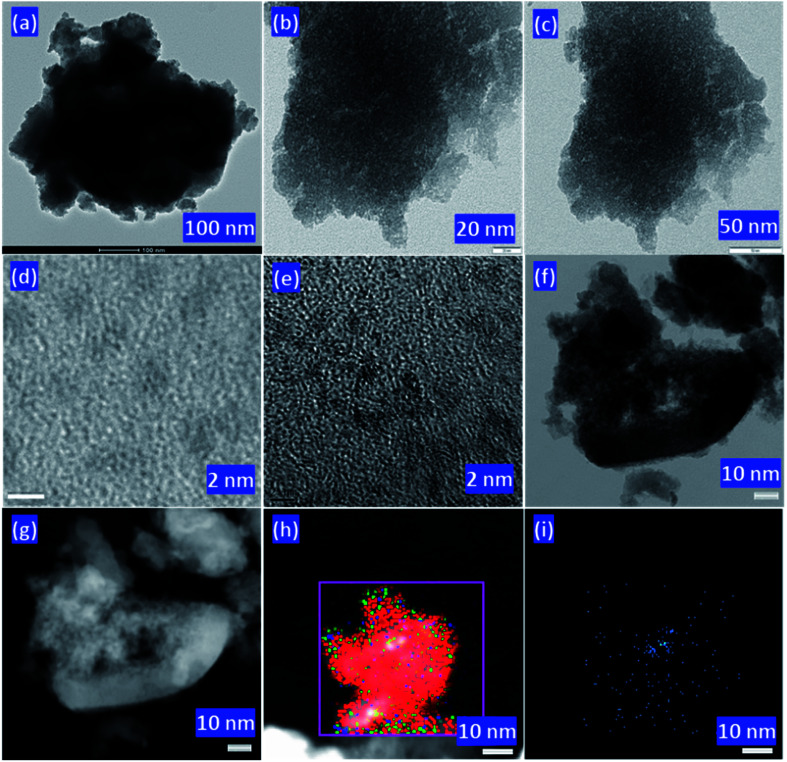
TEM micrographs of PW_11_/ZrO_2_ (a) and Pd–PW_11_/ZrO_2_ (b and c), HRTEM micrographs of Pd–PW_11_/ZrO_2_ (d and e), STEM images bright field (f) & dark field (g) of Pd–PW_11_/ZrO_2_, overlapping image (h) and Pd elemental image (i) of Pd–PW_11_/ZrO_2_ at various magnifications.

**Fig. 5:**
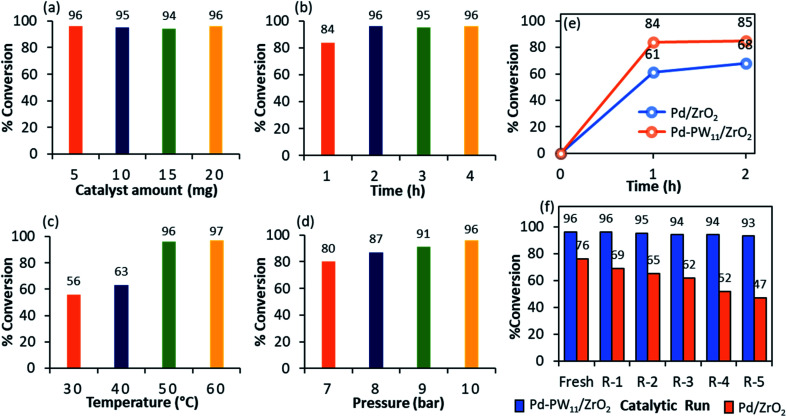
Optimization of reaction parameters: (a) effect of catalyst amount. Cyclohexene (9.87 mmol), H_2_O (50) mL, temp. (80 °C), H_2_ pressure (10 bar), time (4 h); (b) effect of time. Cyclohexene (9.87 mmol), conc. of Pd (0.0034 mol%), H_2_O (50) mL, temp. (80 °C), H_2_ pressure (10 bar); (c) effect of temperature. Cyclohexene (9.87 mmol), conc. of Pd (0.0034 mol%), H_2_O (50) mL, H_2_ pressure (10 bar), time (2 h) and (d) effect of pressure. Cyclohexene (9.87 mmol), conc. of Pd (0.0034 mol%), H_2_O (50) mL, temp. (50 °C), time (2 h). (e) Leaching test: cyclohexene (9.87 mmol), conc. of Pd (0.0034 mol%), substrate/catalyst ratio (29 177/1), H_2_O (50) mL, temp. (50 °C), H_2_ pressure (10 bar), time (2 h). (f) Recyclability test: cyclohexene (9.87 mmol), conc. of Pd (0.0034 mol%), substrate/catalyst ratio (29 177/1), H_2_O (50 mL), temp. (50 °C), H_2_ pressure (10 bar), time (2 h).

TEM images of PW_11_/ZrO_2_ (Fig. 4a) and Pd–PW_11_/ZrO_2_ (Fig. 4b and c) were recorded at various resolution. TEM micrographs of PW_11_/ZrO_2_ show the homogeneous dispersion of PW_11_ over the surface of the ZrO_2_. Selected area electron diffraction (SAED) image of Pd–PW_11_/ZrO_2_ shows the non-crystalline nature of highly dispersed Pd in the synthesized material (Fig. S3†). Whereas, Fig. 5b and c show the high degree of homogeneously dispersed very small isolated PdNPs. To further confirm, HRTEM micrographs were also recorded as shown in Fig. 4d and e, which clearly show the uniform dispersion of PdNPs (particle size, ∼2 nm) throughout the morphology, without aggregates formation, confirming the stabilization of PdNPs by PW_11_. TEM images of Pd/ZrO_2_ are presented in Fig. S3† which show the aggregates formation of Pd(0), indicating the non-stabilized nature of the Pd.

For more insight of PdNPs, an advance technique, STEM was utilized to probe the behaviour of PdNPs. Bright/dark field STEM (BF/DF-STEM) images (Fig. 4f and g, respectively) show the highly dispersed PdNPs all over the morphology of the material. Whereas, overlapping image (Fig. 4h) as well as elemental image of Pd (Fig. 4i) clearly indicates the presence of isolated PdNPs homogeneously dispersed without any cross talks between them. The absence Pd of aggregates conclude that PW_11_/ZrO_2_ is very much capable to decrease the high surface free energy of PdNPs, by providing combinedly the facility of high surface area for dispersion as well as stabilizing nature. More representative images can be found in Fig. S4.†

FT-IR shows the retention of Keggin structure even after impregnation, soaking and post-reduction of the catalyst. XPS confirms the presence of Pd(0) and W(vi). TEM, HRTEM and STEM indicate the sole presence of highly dispersed PdNPs over the surface of the catalyst.

### Catalytic activity

The efficiency of Pd–PW_11_/ZrO_2_ was evaluated towards the hydrogenation. Cyclohexene was selected as the ideal substrate for the reaction to optimize the different influential reaction parameters such as substrate to catalyst ratio (catalyst amount), time, temperature, pressure and solvent for highest product conversion. Reaction scheme is shown in Scheme S1.†

The effect of substrate to catalyst ratio was evaluated by varying the catalyst amount from 5 to 20 mg. Obtained results (Fig. 5a) show that with increasing the catalyst amount from 5 to 20 mg there was no effect on the reaction conversion. Here, very small amount of catalyst *i.e.* 5 mg (0.0034 mol% of Pd) has sufficient active sites and capable to tolerate very high amount of substrate (substrate to catalyst ratio, 29 177/1), clearly indicates the high efficiency of PdNPs.

The influence of time was assessed between 1 to 4 h (Fig. 5b). Initially, up to 2 h, with increasing time, ≈1.14-fold % conversion also increases. Hence, 2 h is the sufficient time for the maximum productive collision of the substrates to yield cyclohexane. Further, increase in time (up to 4 h) has no influence on the % conversion, maximum 96% conversion was achieved in 2 h.

The effect of temperature on reaction was screened in the region of 30–60 °C (Fig. 5c). Obtained results show ≈1.71-fold increase in % conversion with increasing temperature from 30 to 50 °C. Further, rise in temperature shows no appreciable increase in % conversion. At higher temperature there are two facts which resist the reaction conversion: (i) desorption of cyclohexene from the surface of the catalyst and; (ii) lower adsorption rate of hydrogen over the surface of catalyst.^26^ Hence, required activation energy was obtained in just 50 °C for 96% conversion.

The effect of H_2_ pressure was studied in the region of 7 to 10 bar (Fig. 5d). Obtained results show that % conversion increases from 80 to 96% linearly. Hence, the reaction is first order with respect to H_2_ pressure, which is in good agreement with the reported one^27^ stating that hydrogenation is always first order with respect to H_2_ pressure. Further study for effect of high pressure was not carried out as our main focus is to establish environmentally green process. Highest 96% conversion was obtained by applying 10 bar H_2_ pressure.

The optimized conditions for the maximum % conversion (96) with TON (28 010) and TOF (14 005 h^−1^) are: cyclohexene (9.87 mmol), conc. of Pd (0.0034 mol%), H_2_O (50 mL), H_2_ pressure (10 bar), time (2 h) and temp. (50 °C) and substrate/catalyst ratio (29 177/1).

### Control experiments

In order to investigate the role of individual components, control experiments were carried out with ZrO_2_, PW_11_, PW_11_/ZrO_2_, PdCl_2_, Pd/ZrO_2_ and Pd–PW_11_/ZrO_2_ under optimized conditions. Obtained results show that ZrO_2_, PW_11_, PW_11_/ZrO_2_ are totally inactive toward the reaction whereas almost same conversion is achieved by PdCl_2_, Pd/ZrO_2_ and Pd–PW_11_/ZrO_2_ (91, 95 and 96% conversion, respectively). This indicates the Pd is only the responsible real active species for the reaction conversion.

### Leaching and heterogeneity test

In order to investigate the leaching of PdNPs as well as role of PW_11_, the reaction was performed with Pd/ZrO_2_ and Pd–PW_11_/ZrO_2_ up to 1 h, centrifuged to remove the catalyst, and finally the reaction was continued for further 1 h (total 2 h). Organic layer was separated by dicholoromethane, anhydrated by magnesium sulphate and analysed by GC. In case of Pd/ZrO_2_, obtained results (Fig. 5e) indicate the leaching of Pd from the support (conversion, 61% after 1 h and 68% after 2 h). Whereas, Pd–PW_11_/ZrO_2_ shows no rise in % conversion after removal of catalyst [conversion, 84% after 1 h and 85% after 2 h], confirm no leaching of PdNPs during the reaction. To further confirm, the heterogeneity of the Pd–PW_11_/ZrO_2_, the regenerated catalyst was characterized by EDX (Fig. S4†) and reaction mixture by atomic adsorption spectroscopy (AAS). No loss in Pd content (0.71 wt%) indicates the stabilization of PdNPs by PW_11_ over the surface of ZrO_2_. These obtained results confirm the true heterogeneous nature of the catalyst, which is also reflected in recycling study.

### Recyclability and sustainability of the catalyst

Sustainability of the catalyst is the key challenge for any process, and for the same recycling test was studied for Pd/ZrO_2_ and Pd–PW_11_/ZrO_2_ (Fig. 5f). In order to regenerate the catalyst, after reaction completion, the catalyst was centrifuged, washed with dicholoromethane followed by consequent washes with distilled water and finally dried at 100 °C for an hour to reuse it for next catalytic run. Obtained results show the gradual decrease in the % conversion in case of Pd/ZrO_2_, confirm the leaching of active species Pd from the support. Whereas, Pd–PW_11_/ZrO_2_ shows consistent activity up to five catalytic runs. This indicates that there was no emission of PdNPs during the reaction from the surface of the catalyst.

### Characterization of regenerated catalyst

The stability of the regenerated catalyst was studied by its characterization also, such as elemental analysis (EDX), FT-IR, XRD, XPS and HRTEM.

For regenerated Pd–PW_11_/ZrO_2_, the EDX values of Pd (0.71 wt%) and W (15.08 wt%) are in good agreement with the fresh one (0.72 wt% Pd and 15.19 wt% W) confirming no emission of Pd as well as W from the catalyst during the reaction. Elemental mapping is shown in Fig. S5.†

**Fig. 6:**
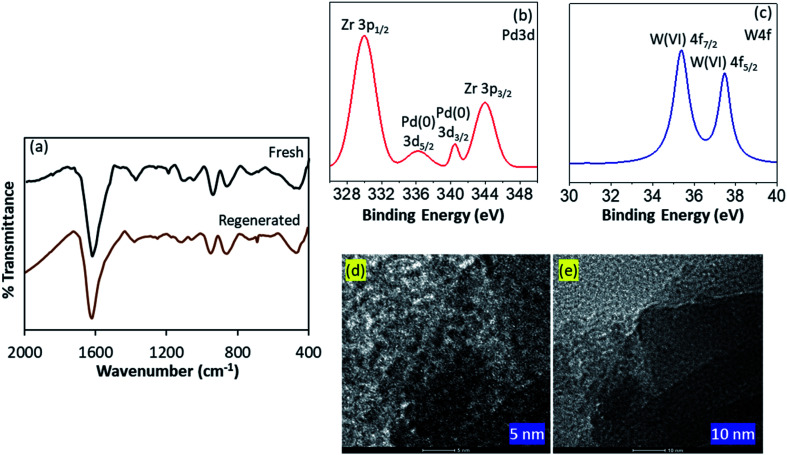
(a) FT-IR spectra of fresh and regenerated catalysts; (b and c) XPS spectra and (d and e) HRTEM images of regenerated catalyst.

FT-IR spectra of fresh and regenerated catalysts are displayed in Fig. 6a. The spectrum of regenerated catalyst was found to be almost identical to fresh one, without any significant shift in the bands. However, the bands intensity for regenerated catalyst was slightly low compared to fresh one, may be due to the sticking of the substrates, which had no effect on the efficiency of the catalyst.

XRD spectra of fresh and regenerated catalyst are shown in Fig. S6.† Obtained results reveal the retention of highly dispersed nature of the catalyst. Absence of any characteristic peaks regarding Pd aggregates as well as PW_11_ clearly indicates the sustainability of the catalyst during the reaction.

XPS spectra of regenerated catalyst is presented in Fig. 6b and c. Almost identical spectra of both fresh as well as regenerated catalysts confirm the retention of Pd atoms over the surface of the catalyst as well as no reduction of W(vi) during the catalytic hydrogenation, assuring the sustainability of the catalyst.

HRTEM images of regenerated catalyst are shown in Fig. 6d and e. Images at various magnification clearly indicates the retention of high dispersion of the isolated PdNPs over the surface of the catalyst. No aggregation of Pd confirms the stabilization of the PdNPs during the reaction *via* PW_11_. More TEM images can be found in Fig. S7.†

For regenerated catalyst, FT-IR indicates the retention of PW_11_ unit although using it for number of consequent catalytic runs. XRD shows the highly dispersed amorphous nature of the catalyst. XPS and HRTEM confirm the presence and homogeneous dispersion of the isolated PdNPs over the surface of the catalyst.

### Scope of catalysis

The scope for hydrogenation of different substrates was also evaluated and the obtained results are enumerated in Table 1.

**Table 1:** Substrate study^a^

Substrate	Product	Conv (%)/sel (%)	TON/TOF (h^–1^)
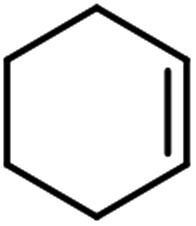	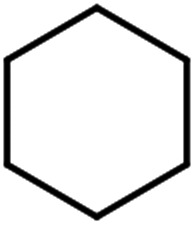	96	28 010/14 005
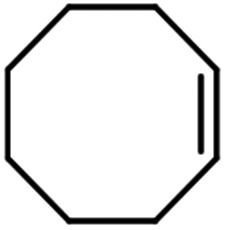	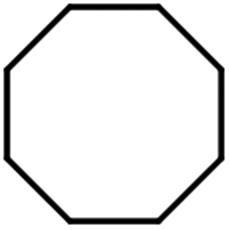	62	18 090/9045
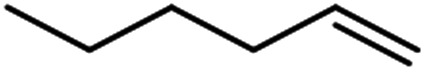	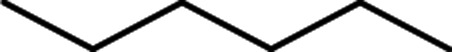	74	21 591/10 780
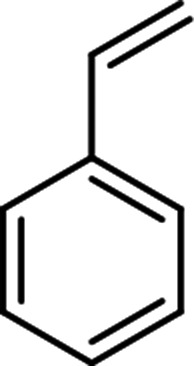	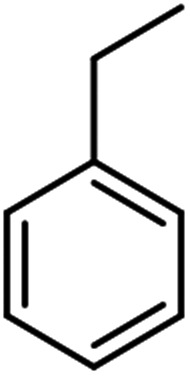	99	28 885/14 443
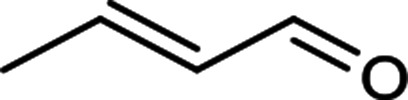	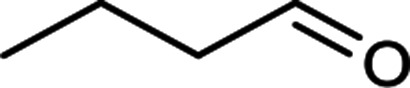	92/100	26 843/13 422
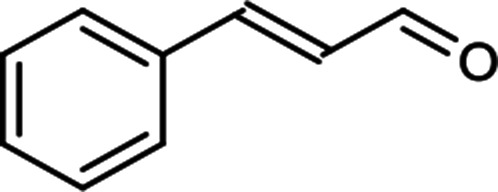	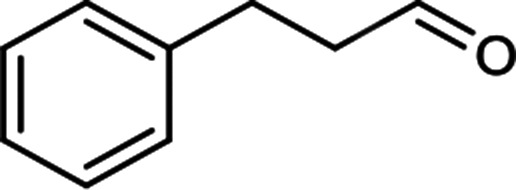	71/100	20 716/10 358

a Reaction conditions: cyclohexene (9.87 mmol), conc. of Pd (0.0034 mol%), H_2_O (50) mL, temp. (50 °C), H_2_ pressure (10 bar), time (2 h).

From the results it is clear that the catalyst is dominantly viable for the selective hydrogenation of C=C including aliphatic and aromatic compounds.

### Comparison with reported systems

The efficiency of the catalyst with reported system is also enumerated in Table 2 with respect to cyclohexene hydrogenation in terms of % conversion, TON as well as TOF. Liu *et al.*^16*a*^ achieved 99% conversion at moderate temperature and very high H_2_ pressure (20 bar) compared to present system. Zhang *et al.*^28^ performed the reaction at low temperature with very poor yield and with high catalyst concentration. Leng *et al.*^29^ reported the use of formic acid as proton transferring agent to achieve 96% conversion using high temperature and catalyst amount compared to the present system. Panpranot *et al.*^16*b*^ obtained 96% conversion utilizing supercritical CO_2_ as a solvent at 60 bar pressure (harsh conditions). Our previously reported system Pd–TPA/ZrO_2_ (ref. 12*d*) was also found to be highly active but required more active amount of Pd as well as higher reaction temperature compared to the present system. Obtain data shows that the present catalyst is best one amongst all reported systems to till date in terms of used catalyst amount, TON as well as TOF under mild reaction conditions.

**Table 2:** Comparison with reported systems in terms of cyclohexene substrate

Catalyst	Pd (mol%)	Solvent	Temp. (°C)	H_2_ pressure (bar)	Conversion (%)	TON/TOF (h^−1^)
SH-IL-1.0wt%Pd^16*a*^	0.02	Auto-clave	60	20	99	5000/5000
Pd/MSS@ZIF-8 (ref. 28)	0.1738	Ethyl acetate	35	1	5.6	560/93
Pd@CN^29^	2.208	Formic acid (proton transfer)	90	—	96	44/4
Pd/SiO_2_ (ref. 16*b*)	0.091	CO_2_ (60 bar)	25	10	96	1097/6582
Pd(0)–TPA/ZrO_2_ (ref. 12*d*)	0.0076	Water	80	10	96	12 604/3151
**Pd**–**PW**_**11**_**/ZrO**_**2**_**(present work)**	**0.0034**	**Water**	**50**	**10**	**96**	**28 010/14 005**

### Effect of addenda atom

In order to check the effect of addenda atom, the reactions were performed using Pd–PW_11_/ZrO_2_ and Pd–PW_12_/ZrO_2_.

(i) *Comparison of TON (% conversion):* hydrogenation was carried out under optimized conditions using both the catalysts and obtained results are enumerated in Table 3. It shows that Pd–PW_11_/ZrO_2_ is highly active (≈6.4-fold %) compared to Pd–PW_12_/ZrO_2_. (ii) *Comparison of TOF:* for more insight, activity of the catalysts was also compared in terms of TOF. For the same, the prolong reactions were carried out under identical conditions to achieve possible maximum % conversion, and the obtained results (Table 3) show that Pd–PW_11_/ZrO_2_ requires only 2 h for fruitful collision of substrates to achieve maximum 96% conversion with TOF 14 005 h^−1^ whereas, Pd–PW_12_/ZrO_2_ requires 8 h for 97% conversion with TOF 3538 h^−1^.

**Table 3:** Effect of addenda atom on conversion/TON/TOF^a^

Catalyst	Conversion (%)/TON^b^	Time (h)/Conv%/TOF (h^−1^)	Number of protons
Pd–PW_12_/ZrO_2_	15/4377	8/97/3538	3
Pd–PW_11_/ZrO_2_	96/28 010	2/96/14 005	7

a Reaction conditions: cyclohexene (9.87 mmol), conc. of Pd (0.0034 mol%), substrate/catalyst ratio (29 177/1), H_2_O (50 mL), temp. (50 °C), H_2_ pressure (10 bar).

b Time (2 h).

Here, high activity of Pd–PW_11_/ZrO_2_ is attributed to greater number of counter protons compared to Pd–PW_12_/ZrO_2_. It is well known that for surface phenomenon type catalytic hydrogenation, the formation of Pd–H is necessary, higher the formation of Pd–H more the % conversion. As Pd–PW_11_/ZrO_2_ consists seven hydrogen (in the form of counter protons), which accelerate the formation of Pd–H to enhance the hydrogenation rate, whereas Pd–PW_12_/ZrO_2_ has only three counter protons and as result it activates the reaction moderately.

### Mechanistic study

The mechanistic study was investigated by carrying out, (i) control experiments, (ii) reaction in inert atmosphere and (iii) reaction using D_2_O as solvent.

**Fig. 7:**
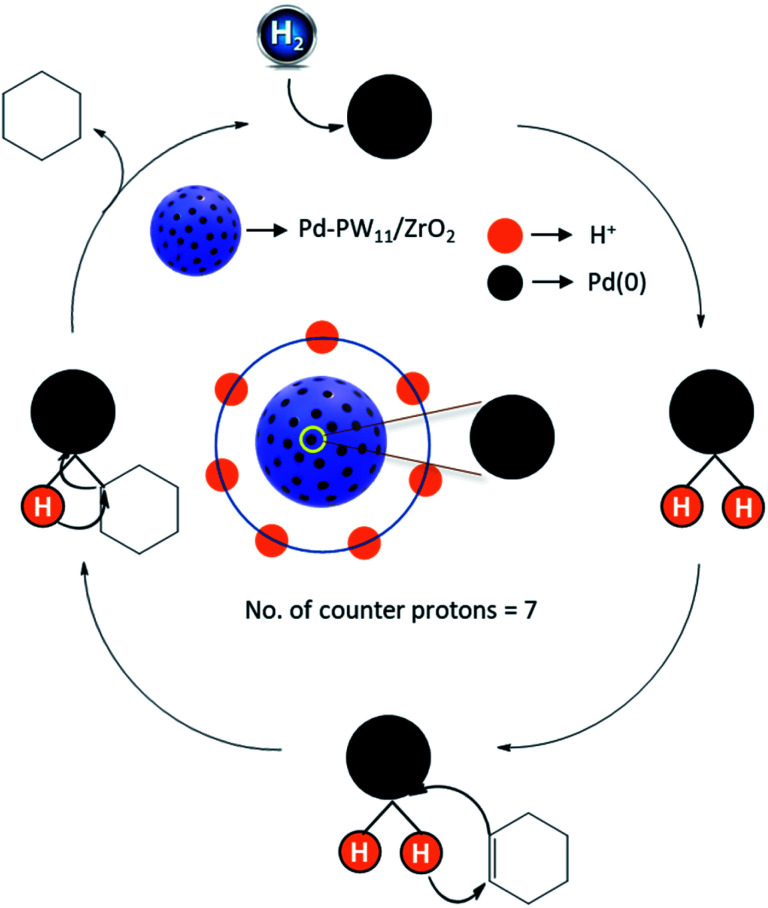
Proposed reaction mechanism.

(i) *Control experiments:* to study the roll of each component as well as the responsible active species, the reaction was performed using ZrO_2_, PW_11_, PW_11_/ZrO_2_, PdCl_2_ and Pd–PW_11_/ZrO_2_ under optimized conditions. Achieved results (as shown in control experiment study) indicate that Pd is only the active sites for the reaction. (ii) *Inert atmosphere:* to ensure the hydrogenation *via* molecular H_2_, the reaction was performed under N_2_ pressure instead of H_2_ and no conversion assures the necessity of H_2_ for reduction. (iii) *D_2_O as solvent:* to investigate the role of water, reaction was performed using D_2_O as solvent, no formation of deuterated product (cyclohexane, confirmed by ^1^H NMR) exposed that the hydrogen is directly transferred from molecular H_2_, not from water. These results are in good agreement with conventional mechanism for hydrogenation of unsaturated hydrocarbon. Hence, we are proposing the same mechanism in which mechanism runs by formation of palladium hydride formation *via* homolytic cleavage of H_2_ molecule, followed by hydrogen transfer to the unsaturated bond. Based on this data, Fig. 7 shows the proposed mechanism.

## Conclusions

In this article, first time we report the synthesis of stabilized PdNPs *via* supported mono lacunary tungstophosphoric acid (Pd–PW_11_/ZrO_2_) by wet chemistry method. The presence of PdNPs as active sites was exposed by HRTEM, BF & DF-STEM analysis. The efficiency of the catalyst was evaluated for hydrogenation of cyclohexene as a model substrate, superiority of the present work lies in obtaining high conversion (96%) with high substrate/catalyst ratio (29 177/1) using H_2_O as a green solvent at just 50 °C. The established TON (28 010) as well as TOF (14 005 h^−1^) is the highest one to till date for cyclohexene hydrogenation. The present catalyst can be reused up to number of cycles without any degradation or leaching and found to be viable under sustainable reaction conditions to tolerate variety of the substrates. Correlation of catalysts activity with effect of addenda atom prove the surpassing activity of newly designed present catalyst compared to our previously reported catalyst (Pd–PW_12_/ZrO_2_) and open the door for engineering of number of efficient catalysts using different polyoxometalates. We believe that present strategy can be extended for the designing of other precious metal-based NPs for various organic transformations.

## Author contributions

Anish Patel: Conceptualization, formal analysis, investigation, methodology, supervision, visualization, writing–review & editing. Anjali Patel: Conceptualization, supervision, writing–review & editing. The manuscript was written through contributions of all authors. All authors have given approval to the final version of the manuscript.

## Conflicts of interest

There are no conflicts to declare.

## Supplementary Material

RA-011-D1RA00239B-s001
