# Supplementary material for: Synthesis and characterization of supported stabilized palladium nanoparticles for selective hydrogenation in water at low temperature
Source: RSC Adv. 2021 Feb 19;11(14):8218–27. doi: 10.1039/d1ra00239b (PMC8695083; doi:10.1039/d1ra00239b)
Supplement: RA-011-D1RA00239B-s001 [file RA-011-D1RA00239B-s001.pdf]

Electronic Supplementary Material (ESI) for RSC Advances.
This journal is © The Royal Society of Chemistry 2021

Supplementary Information

Synthesis and Characterization of Supported Stabilized Palladium Nanoparticles for Selective Hydrogenation in Water at Low Temperature

Anish Patel and Anjali Patel*

Polyoxometalates and Catalysis Laboratory, Department of Chemistry, Faculty of Science.

The Maharaja Sayajirao University of Baroda. Vadodara-390002. Gujarat. India.

*E-mail: anjali.patel-chem@msubaroda.ac.in

ORCID: 0000-0002-6871-7564 and 0000-0003-0177-3956*

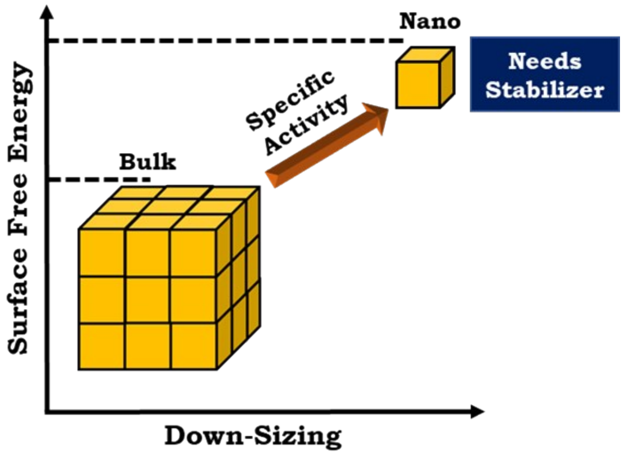

Figure S1 Nanosizing of catalyst from bulk and associated surface free energy.

Catalyst Synthesis

Sodium salt of mono lacunary tungstophosphoric acid (Na_7PW_{11})

Sodium tungstate dihydrate (0.022 mol, 7.2 g) and anhydrous disodium hydrogen phosphate (0.002 mol, 0.284 g) were dissolved in 15–20 mL of distilled water and heated to 80 °C followed by the addition of diluted nitric acid to adjust the pH to 4.8. The volume was then reduced to half by evaporation and the heteropoly anion was separated by liquid–liquid extraction with 80-100 mL of acetone. The extraction was repeated until the acetone extract showed the absence of NO_3^- ions (ferrous sulfate test). The extracted sodium salt was dried in air. The resulting material was designated as Na_7PW_{11}.

Synthesis of Zirconia (ZrO_2)

Aqueous ammonia solution was added to aqueous solution of $ZrOCl_2{\cdot}8H_2O$ (10 % w/v) up to pH 8.5. The precipitates were aged at 100 °C on the water bath for 1 h, filtered, washed with conductivity water

until chloride free filtrate was obtained and dried at 100 °C for 10 h. The obtained material was designated as ZrO_2.

Synthesis of PW_{11}/ZrO_2

PW_{11}/ZrO_2 was synthesized by wet impregnation method. 1 g of ZrO_2 was impregnated with an aqueous solution of Na_7PW_{11} (0.3/30 g mL^{-1} of double distilled water) at 100 °C followed by drying at same temperature in oven for 10 h. The obtained material (Na_7PW_{11}/ZrO_2) was protonated with 0.01 N HCl, filtered, washed with double distilled water and dried at 100 °C. The obtained material was designated as PW_{11}/ZrO_2.

Synthesis of Pd/ZrO_2

1 g of ZrO_2 was soaked with 25 mL of 0.05 M aqueous solution of $PdCl_2$ for 24 h with stirring. The solution was filtered, washed with distilled water in order to remove the excess of $PdCl_2$ and dried in air at room temperature. The resulting (brown colored) material was designated as Pd(II)/ZrO_2. Finally, the synthesized material was charged in a Parr reactor under 1 bar H_2 pressure, at 40 °C for 30 min to reduce Pd(II) to Pd(0). The obtained (black colored) material was designated as Pd/ZrO_2.

Characterization

The amount of Pd as well as W was determined by gravimetric method [1]. Energy dispersive X-ray spectra and hyperspectral element maps were acquired by JSM 5610 LV combined with INCA instrument using carbon strip. Thermo gravimetric analysis (TGA) was performed using Mettler Toledo Star SW 7.01 up to 500 °C under nitrogen atmosphere. Nitrogen physisorption isotherms were measured through Micromeritics ASAP 2010 Surface area analyzer at -196 °C. Specific surface area was calculated using Brunauer-Emmett- Teller (BET method). The samples were degassed at 75 °C for 7 h prior to analysis. Leaching of Pd in the reaction mixture was checked by using atomic adsorption spectrometer AAS GBC-902 instrument. FT-IR spectrum of the materials were performed by using the KBr wafer on Shimadzu instrument (IRAffinity-1S). Powder X-ray Diffraction (Powder XRD) was carried out using Philips Diffractometer (Model PW-1830). X-ray photoelectron spectroscopy (XPS) measurements were performed with Auger Electron Spectroscopy (AES) Module PHI 5000 Versa Prob II. Transmission electron microscopy (TEM) micrographs were recorded on TEM CM 200 (Make: PHILIPS, Model: CM 200, Specification: Operating voltages: 20-200kv and Resolution: 2.4 Å). High-resolution transmission electron microscopy (HRTEM) analysis were carried out on Field Emission Gun-Transmission Electron Microscope (Resolution: Point: 0.19 nm, Line: 0.1 nm, Magnification: 50 x - 1.5 M x accelerating voltage of 200 kV; Make: JEOL; Model: JEM 2100F) with attachment systems EDS and STEM. The samples were dispersed in ethanol and ultrasonicated for 5–10 min. A small drop of the sample was then taken on a carbon coated copper grid and dried before viewing. It must be noted

that Pd(0)/ZrO_2 has not been characterized in detail as the main focus was on the comparison of the catalytic activity.

Results and Discussion

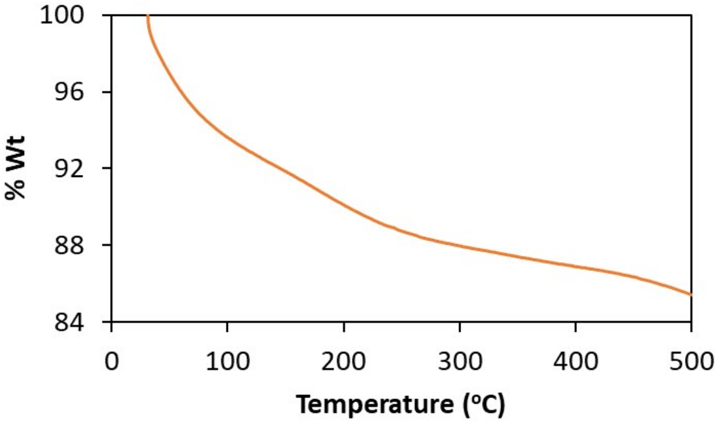

Figure S2. TGA curve of Pd-PW_{11}/ZrO_2.

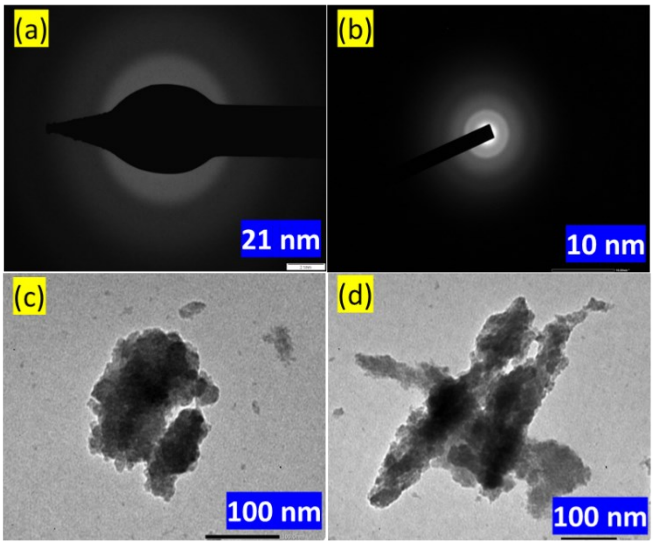

Figure S3. SAED images of (a) Pd-PW_{11}/ZrO_2, (b) Pd/ZrO_2 and (c & d) TEM images of Pd/ZrO_2.

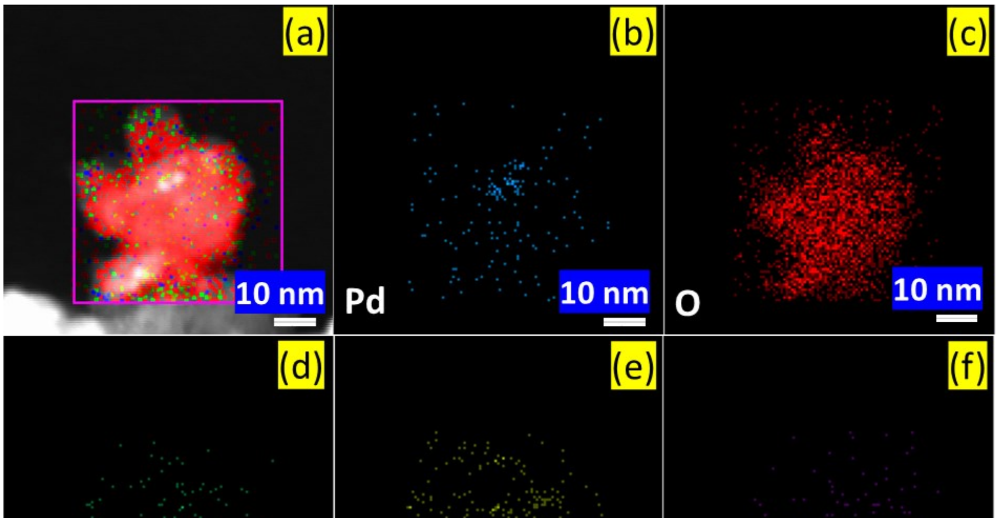

Figure S4. (a) Overlapping image and (b-f) elemental images of Pd-PW_{11}/ZrO_2.

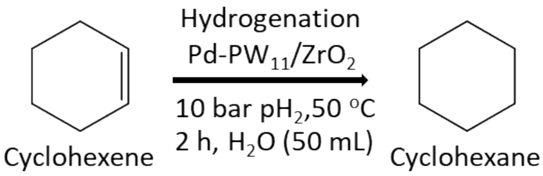

Scheme S1. Cyclohexene hydrogenation.

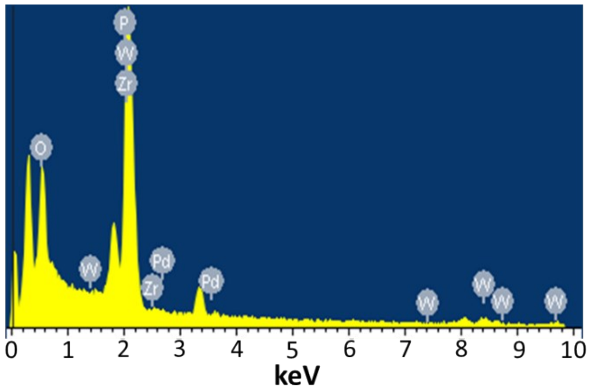

Figure S5. EDS elemental mapping of regenerated catalyst.

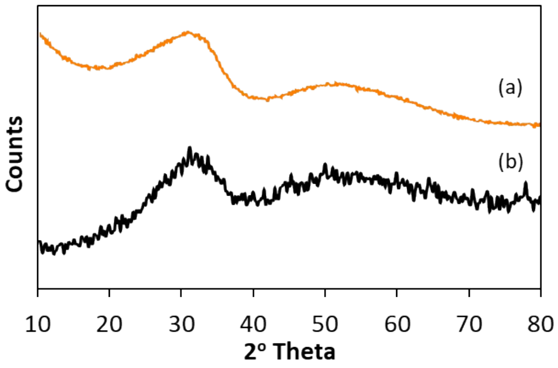

Figure S6. Powder XRD spectra of (a) fresh and (b) regenerated catalysts.

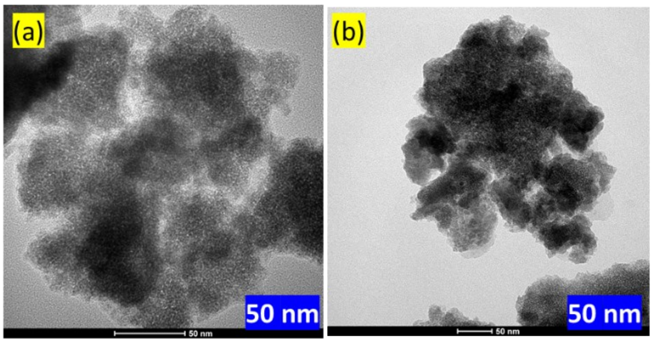

Figure S7. TEM images of regenerated catalyst.

Reference

[1] Vogel, A. I. & Jeffery, G. H. Vogel's textbook of quantitative chemical analysis. (Longman Scientific & Technical, 1989).
